# Messrs. Scott and Taynton, on Vaccination

**Published:** 1806-03

**Authors:** 

**Affiliations:** Surgeon. Bromley, Kent; Surgeon. Bromley, Kent


### *To the Editors of the Medical and Physical Journal.*

GENTLEMEN,

THE small-pox, for the last three or four months has been unusually prevalent in the town of Bromley, and the adjoining village; and as great numbers of the children in this neighbourhood were vaccinated about three or four years ago, the prophylactic efficacy of the cow-pox has been put to a very decided test. It has frequently happened that children who were vaccinated at a former period, have, in a small room, and for a considerable time, breathed an atmosphere, rendered highly infectious by the respiration of other children of the same family labouring under the variolous disorder. You will readily conceive, that under these circumstances, various reports, originating in ignorance and prejudice, have been circulated to the disadvantage of vaccination. In fact, the same arts have been practised here, which have disgraced many other places, and particularly the metropolis, to discourage people from availing themselves of this most fortunate discovery; a discovery which will hand down the name of the immortal Jenner to the remotest posterity, as the greatest benefactor of his species. These reports have produced a lukewarmness in some of the friends of vaccination, an unwillingness in the poor to have their children vaccinated; and have infused doubts into the minds of many persons, who from their education, and more liberal habits of thinking, were certainly competent to make a more correct estimate of that cloud of evidences in favour of its prophylactic virtue, which from the commencement of the discovery to the present moment, have been perpetually accumulating, not only in this kingdom but in all parts of the world. We have beheld the effects of these unfounded rumours rumours with a very lively concern; and it is a duty which we owe to the public, to enter our most solemn protest against them; and to declare thus openly and confidently, that we have witnessed only *a single exception*, (which shall immediately be explained) and that no other individual, who had been vaccinated at any former period, has taken the small-pox, though under circumstances in which it was impossible to have escaped the infection, had they not been secured by the mild, but unquestionable influence of vaccination.

### CASE.

Henry Cooper, (according to our case-book, from which we have made the following extract) was vaccinated at the age of four months, on March 15, 1800. The arm rose, and the pustule went through the regular stages; but the surrounding efflorescence was much less than usual.' Early in January last, this child was attacked with fever, which was followed by a considerable eruption. The pustules were in general smaller than they are in the natural small-pox, but some of them certainly bore the variolous character. This child's brother, who never had been vaccinated, died at the same time of the confluent small-pox.

In this case the absence of a due quantity of inflammation surrounding the pustule, was a remarkable peculiarity; and from our noticing it in this particular instance *at the time*, and not finding a similar remark on any *other of our* cases, which we *recorded*, it is obvious to conclude, that we *did* entertain some doubts of this child's security.

We have deemed this case deserving of publication, because a general distrust in the preventive powers of vaccination has been excited by it in this neighbourhood. Whatever may be the public opinion respecting this case, it is after all but a *solitary one*; and *our* confidence in vaccination is not diminished by it. We most sincerely lament the prejudices to which it has given rise; and *we* are determined strenuously to support the *vaccine* inoculation, and oppose the *variolous*. It is much in the power of *professional men* successfully to combat such prejudices; and it is their *duty*, we apprehend, to eradicate them, *if possible*, from the minds of the public.

We remain, &c.

SCOTT & TAYNTON, SURGEONS.

*Bromley, Kent, February* 12, 1806.

